# Electrotunable Lubricity with Ionic Liquid Nanoscale Films

**DOI:** 10.1038/srep07698

**Published:** 2015-01-09

**Authors:** O. Y. Fajardo, F. Bresme, A. A. Kornyshev, M. Urbakh

**Affiliations:** 1 School of Chemistry, Tel Aviv University, 69978 Tel Aviv, Israel; 2 Deparment of Chemistry, Imperial College London, SW7 2AZ London, U.K

## Abstract

One of the main challenges in tribology is finding the way for an *in situ* control of friction without changing the lubricant. One of the ways for such control is via the application of electric fields. In this respect a promising new class of lubricants is ionic liquids, which are solvent-free electrolytes, and their properties should be most strongly affected by applied voltage. Based on a minimal physical model, our study elucidates the connection between the voltage effect on the structure of the ionic liquid layers and their lubricating properties. It reveals two mechanisms of variation of the friction force with the surface charge density, consistent with recent AFM measurements, namely via the (i) charge effect on normal and in-plane ordering in the film and (ii) swapping between anion and cation layers at the surfaces. We formulate conditions that would warrant low friction coefficients and prevent wear by resisting “squeezing-out” of the liquid under compression. These results give a background for controllable variation of friction.

Ionic Liquids (ILs) are molten salts with melting points below 100°C, whose physical properties can be controlled through systematic changes in the molecular structure of the cation-anion pairs^1^. ILs have recently attracted considerable interest as lubricants due to their physical properties: negligible vapor pressures, high temperature stability and high ionic conductivity^2^,^3^,^4^. ILs interact strongly with solid surfaces^5^ and can act as wear-protective films for normal loads that are much higher than those of existing molecular lubricants^6^. Recent studies of macroscale friction in ILs have demonstrated the possibility of tuning the lubricity by varying the IL composition^2^,^3^,^4^. Understanding of the molecular origin of the observed changes in the friction response remains challenging.

The need to find the molecular mechanism of friction in ILs has led to a number of experimental studies in nanoscale confinement, employing both the surface force apparatus (SFA)^7^,^8^ and atomic force microscopy (AFM)^9^,^10^,^11^,^12^,^13^. At the nanoscale, ILs embedded between charged surfaces feature alternating positive and negative ion layers, with an interlayer separation that corresponds to the ion pair size^6^,^7^,^9^,^13^,^14^,^15^,^16^,^17^,^18^,^19^. In contrast to molecular liquids, ILs squeeze out two layers at a time, one cation layer and one anion layer, in order to maintain system electroneutrality. Distinct friction regimes for films consistent of 3 to 9 ion layers have been observed. These films feature the so called “quantized friction”, a discrete multi-valued friction behaviour as a function of the load and the number of the confined ionic layers^7^. The friction force was found increasing with decreasing number of the layers^7^,^9^,^13^.

A unique path to controlling and ultimately manipulating lubricating properties of ILs confined between solid surfaces is through an application of an electric potential to the sliding contact. This can be achieved in friction experiments performed under electrochemical conditions, utilizing the fact that IL is an electrolyte. In such systems if surfaces are conductive, their potentials can be independently varied relative to a reference electrode in the bulk, as it was done in AFM measurements. In existing SFA experiments with mica walls^6^,^7^,^8^, the latter are spontaneously negatively charged due to dissociation of surface K^+^ ions into the surrounding IL, however so far no direct control or characterization of the surface charges was achieved. Generally, if the walls are not conductive, electric field across the nanogap can still be imposed and controlled by backing the walls with metallic plates, and applying large voltage between the plates^20^. This has yet been demonstrated in polyelectrolytes but not ILs.

AFM measurements in an IL at the Au(111) electrode^11^ reported a modification of the friction forces, possibly connected to potential-induced changes in the composition of a confined ion layer between the two surfaces, from cation-enriched (at negative potentials) to anion-enriched (at positive potentials). AFM experiments in ILs confined between the silica tip and graphite surface^12^ demonstrated that super-low friction (superlubricity) can be “switched” on and off in situ, by polarizing the surface relative to the reference electrode. Thus, for a given lubricant and under given tribological conditions (normal load, pulling velocity and temperature) the nanoscale friction can be efficiently tuned via application of electric field to either increase or decrease lubricity. Importantly, the response to a potential change depends on the IL used. This opens a new pathway to achieve desirable friction properties.

Unravelling the mechanism of nanoscopic friction in ILs and its relation to the fluid's structure still poses a great scientific challenge, and so far very few studies in this direction have been performed^21^,^22^,^23^,^24^,^25^. The results obtained demonstrated that the shape of IL molecules may affect their layering structure, and pointed out the strong influence of in-plane ordering on friction. In spite of the first successful experimental demonstrations of controling friction in IL through an application of an electric potential, so far there have been no theoretical or computational studies of this effect. The key questions had still to be answered: (1) What are the fundamental mechanisms behind the observed variation of friction with the electrostatic potential? (2) In which systems a significant reversible variation of friction with potential can be achieved? (3) Under which conditions vanishingly low level of friction can be reached?

## The model and simulation strategy

**Figure 1:**
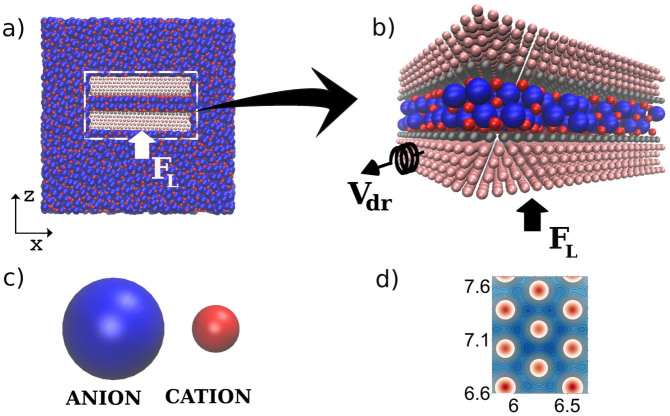
The sketch of the system. (a) Side view of the typical configuration of the system under normal loading in the *z*-direction and sheared in *y*-direction. (b) Zoom of the confined region showing the IL confined between two walls. The upper wall is fixed and the bottom wall is loading with the force *F_L_* and pulling through the spring of stiffness, *K_dr_* = 16 N/m, at constant velocity *V_dr_* = 10 m/s. The dimensions of the walls are 7.56 nm and 7.95 nm in x and *y* directions, respectively, and the size of the atoms is *σ_w_* = 0.3218 nm. (c) The diameters of the cations and anions are *σ_c_* = 0.35 nm and *σ_a_* = 0.70 nm, respectively. (d) Potential energy surface for cation sliding in *x*, *y*-directions along the surface, distances displayed in nm. Maxima and minima of the potential are shown by red and blue colors, respectively.

In this article we propose a minimal model for the description of the effect of an electric field on nanoscopic friction mediated by ILs. ILs have been modeled as a 1 to 1 mixture of oppositely singly charged spheres interacting through short-range repulsive Lennard-Jones, 4*ε_i−i_*/(*r*/*σ_i−i_*)^12^, and coulombic potentials. Coarse grained simulation of generic features of ILs provided a clear insight into the effects of overscreening and crowding at surfaces and in nanogaps^26^,^27^,^28^,^29^, and we adopt a similar approach here. We set the diameter of the spheres to σ_c_ = 0.35 nm for cations and σ_a_ = 0.70 nm for anions, in order to explore the effect of asymmetry of ion sizes (the case when cations are larger than anions can be studied in a similar way). Such Molecular Dynamics (MD) simulations were performed for a mixture consisting of 5,412 cations and 5,412 anions interacting with two atomistic parallel walls in the x–y plane mimicking mica surfaces (see Fig. 1). Each wall consisted of five (111) planes of an fcc lattice and included 2,838 Lennard-Jones spheres. Short range interactions between the IL and the wall atoms were described by Lennard-Jones potentials including both repulsive and attractive contributions. It should be noted that the cation radius was taken close to the radius of the wall atoms, while the radius of anions was twice as large. For further simulation details, see Methods.

In simulations we controlled the charge on the surfaces, by assigning a partial charge to each wall surface atom. In fact, when dealing with conducting surfaces, experimentally one controls the potential to which the charge distribution on the surfaces is adjusted. Hence, for each surface charge density, *q_s_*, one can calculate the surface potential with respect to the bulk of IL, as well as the potential distribution inside the nanogap. The electrostatic potentials, which emerge in our simulations, lie within the range of one Volt.

In the present article, we focus on the effect of the surface charge density, *q_s_*, on the layering and friction, while the systematic studies of the effect of normal load on friction will be reported eslewhere. The walls were held together by a constant normal load, *F_L_*, applied in the z-direction. In our simulations the particles of one of the walls were kept fixed by using a restraining potential, while the other wall could slide by pulling it along the y direction at constant velocity *V_dr_*, The pulling was performed via a spring of stiffness *K_dr_*. In all our simulations the system exhibits irregular stick-slip motion.

## Results and Discussion

### Friction between identically charged surfaces

Inspired by the reported SFA measurements^6^,^7^,^8^, we first consider ILs confined between identically negatively charged walls. Our simulations demonstrate that in this case the ILs arrange themselves into an odd number of layers with alternating positive and negative charges. The results presented in this paper correspond to the IL with five confined layers. We obtained similar results for three and seven layers (not shown). In agreement with the experimental observations^7^,^9^,^13^, for given normal load and pulling velocity the calculated friction force decreases with the increase of the number of layers.

**Figure 2:**
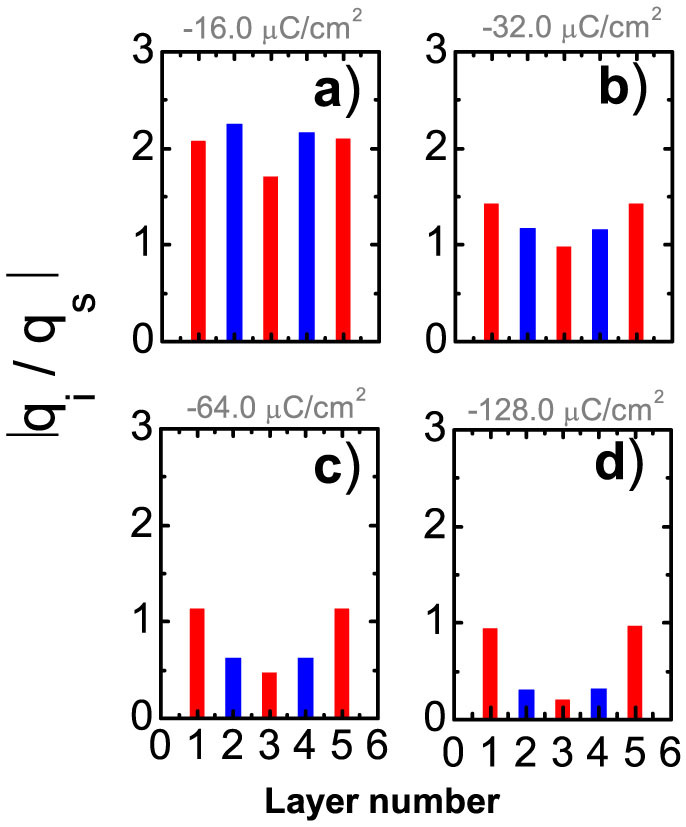
Overscreening effect in ionic liquid confined between equally charged walls. Calculated charge densities per unit surface area in cation(red)- and anion (blue)-rich layers normalized by the surfaces charge density, *q*_s_. The results are shown for four different values of *q*_s_: (a) −16 μC/cm^2^, (b) −32 μC/cm^2^, (c) −64 μC/cm^2^, (d) −128 μC/cm^2^. In all cases *F_L_* = 188 MPa and *V_dr_* = 10 m/s.

In Fig. 2 we show the effect of *q_s_* on the charge density of the IL confined layers. The layers were numbered starting from the one next to the fixed wall. The density of the ions in the first and fifth layers increases with *q_s_*, while the densities in the inner layers change only slightly. Figure 2 shows a dramatic overscreening at small and medium surface charges. Indeed, the layers in direct contact with the walls include more countercharges than are present on the surfaces, and adjacent layers then feel a smaller net charge of opposite sign, which is again overscreened. Increasing the surface charge density leads to a gradual reduction of the overscreening effect in favor of the formation of the condensed layers of counterions. The effect of overscreening at low surface charges (voltages) is a generic feature of ILs at charged interfaces that has been observed experimentally and found in recent simulations^26^,^27^ and theory^30^,^31^. As we show below, overescreening strongly influences the charge dependent friction forces in nano-confined ILs.

**Figure 3:**
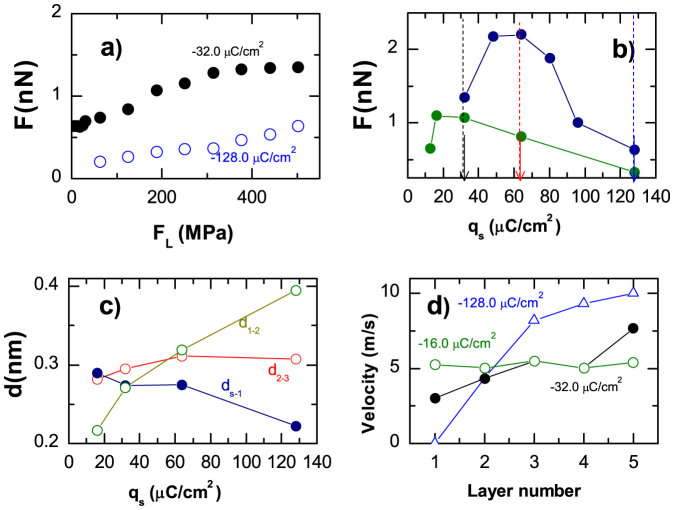
Rationalizing friction between equally charged surfaces. (a) Loading effect on friction shown for two indicated surface charge densities, *q_s_* = −32 μC/cm^2^ and *q_s_* = −128 μC/cm^2^. (b) Nonmonotonic charge dependencies of the friction force, shown for two loads, *F_L_*: 188 MPa (green curve) and 500 MPa (blue curve). (c) Charge dependence of the mean distances between the wall and the first layer (blue curve), the first and second layers (green curve) and the second and third layers (red curve). (d) Velocity profiles inside the IL film for three values of surface charge density *q_s_* : −16 μC/cm^2^ (open circles), −32 μC/cm^2^ (filled circles) and −128 μC/cm^2^ (triangles); shown for *F_L_* = 188 MPa.

The results presented in Figs. 3a–b show a strong dependence of the time-averaged friction force on the surface charge density. For a given normal load the friction force can be decreased by an order of magnitude by varying *q_s_*_._ In agreement with experimental observations^11^,^12^ we found a low friction force with a friction coefficient of ≈0.02 for high surface charge densities. However, the friction force dependence on the surface charge density is nonmonotonic (see Fig. 3b). The data are shown in the interval of *q_s_* for which the system exhibits five IL layers. For smaller absolute values of *q_s_* ([*q_s_*] < 10 *μC*/*cm*^2^], at *F_L_* = 188 MPa, and |*q_s_*| < 30 *μC*/*cm*^2^, at *F_L_* = 500 MPa) we observed a transition to three layers.

In order to understand the origin of the friction-charge dependence, we computed the distances between the fixed wall and the adjacent (first) layer of the IL, *d_S_*_-1_, and between the first and second IL layers, *d*_1–2_, as a function of the surface charge density (see Figs. 3c). The distances *d_S_*_–1_ and *d*_1–2_ exhibit opposite trends with increasing *q_s_*. While *d_S_*_–1_ decreases due to an enhancement of the electrostatic attraction between the wall and the first layer, *d*_1–2_ increases, since the overscreening is reduced and the attraction between the second and the first layers becomes weaker, whereas entropy tends to expand the film. The distance between the second and third layers slightly increases with charge in the range of small |*q_s_*| (*q_s_* > −30 *μC*/*cm*^2^), saturating at large |*q_s_*| because of the finite load.

The charge-induced *decrease* of *d_S_*_-1_ enhances the corrugation of the potential energy landscape (see Fig. 1d) experienced by the first layer of ions during sliding. Simultaneously, charge-induced *increase* of *d*_1–2_ reduces the corrugation potential between the first and second layers. Correspondingly, with the increase of |*q_s_*| the friction between the first layer and the wall grows, while the friction between the first and the second layers decreases.

The interplay between these two opposite trends results in the nonmonotonic dependence of the overall friction force on the surface charge density, shown in Fig. 3b. Indeed, the variation of the interlayer distances with surface charge affects the velocity profiles of the IL films (see Fig. 3d). For low values of |*q_s_* | the electrostatic attraction between the walls and the adjacent layers of IL is relatively weak, and the main slippage occurs at the interfaces between the walls and the adjacent cation layers. In this case all the layers of IL slide with approximately the same average velocity, which corresponds to half of the pulling velocity, *V_dr_*. Considering that the shear stress and, accordingly, the friction force between the IL layers are proportional to their relative velocities^32^, we can conclude that for low surface charge densities the energy dissipation mainly occurs between the walls and the adjacent cation layers. Thus, for surface charges corresponding to the ascending side of the *F*(*q_s_*) curve in Fig. 3b, the overall friction force is dominated by the friction at the interfaces between the walls and adjacent cation layers. The latter grows with increasing |*q_s_*|, and correspondingly *F* increases. With further increase of |*q_s_* | the first and fifth layers stick to the corresponding walls and the dominant slippage and energy dissipation take place at the interfaces between the 1^st^ and 2^nd^, as well as 4^th^ and 5^th^ layers. With *F*(*q_s_*) dominated then by the friction at these interfaces, which itself diminishes as *d*_1–2_ and *d*_4–5_ increase with |*q_s_* |, the average friction force decreases with *q_s_*.

Thus, our simulations show that at high surface charges ILs behave as “ideal” lubricants: they (i) have low friction coefficient, and (ii) resist “squeeze-out” under compression, which prevents wear, due to strong attachment to the walls of the adjacent ionic layers.

**Figure 4:**
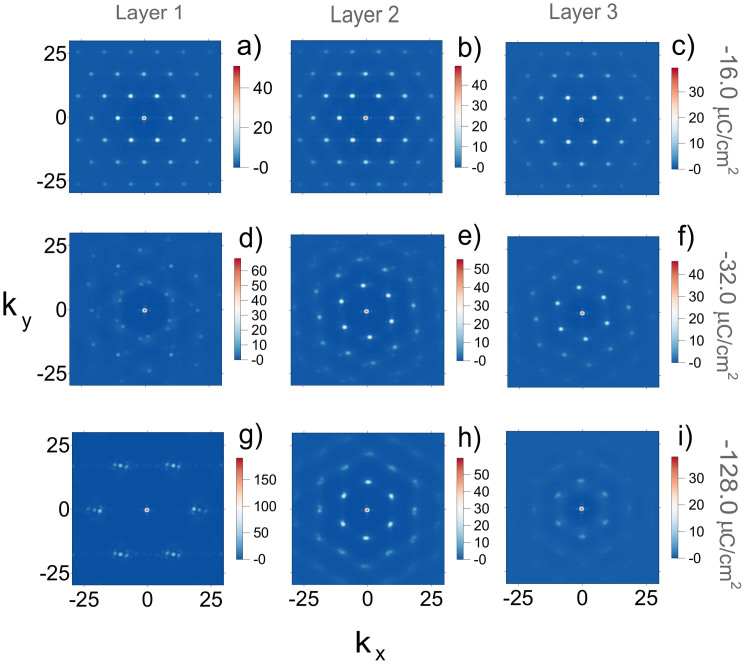
Lateral order in ionic layers between equally charged surfaces. Structure factors *S_m_*(*k*) for the first three IL layers calculated for three indicated values of surface charge density, *q_s_*: −16 μC/cm^2^, −32 μC/cm^2^, and −128 μC/cm^2^. Increase of the surface charge induces inncommensurate-to-commensurate transition in the first layer.

Previous studies of confined liquids have demonstrated that the in-plane ordering in the liquid films and the commensurability of the walls and adjacent liquid layers play a key role in defining the transmission of the shear stress across the interface^33^,^34^,^35^. To give a quantitative measure of the in-plane ordering we calculated the time-averaged two-dimensional structure factors, *S_m_(k_x_,k_y_*), for each IL layer (*m* = 1, …, 5), corresponding to three different surface charges. The structure factor is defined as 
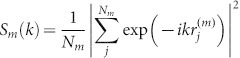
, where 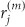 are the coordinates of ions in *m*-th layer and *N_m_* is a number of ions in this layer. The simulations show that *S_m_* as a function of *m* is almost symmetric with respect to the middle layer (*m* = 3), hence we show in Fig. 4 the patterns of *S_m_* for the first three layers. It demonstrates that the magnitude of the surface charge density strongly influences the in-plane ordering in the ionic layers adjacent to the walls, but it has only a minor impact on the lateral structure of the inner liquid layers.

For small surface charge densities, *q_s_* = −16 *μC*/*cm*^2^, the structure factors for all the layers are almost identical. They are consistent with hexagonal ordering in the layers, which is driven by the electrostatic repulsion between the ions. Thus, here we find commensurate structures between the liquid layers, and incommensurate contacts between the IL and the walls. These results indicate that the IL slides as a whole with respect to the walls, which is reflected in the flat velocity profile shown in Fig. 3d.

Upon increasing |*q_s_*| the densities of ions in the first and fifth layers increase. As a result the structure factor, *S*_1_(*k*), starts to display features corresponding to the honeycomb lattice due to the interaction with the wall. At surface charge densities, as large as |*q_s_*| > 50 *μC*/*cm*^2^, the lateral structures of the first and fifth layers are completely determined by the interaction with the walls. These layers are commensurate with the wall surfaces and incommensurate with the inner IL layers.

Thus, the variation of the surface charge density influences the ordering of the confined ILs in both normal and lateral directions, and in both cases the increase of |*q_s_*| leads to the shift of the slippage plane from the solid-liquid interface to the interface between liquids layers (see Fig. 3c).

### Friction between oppositely charged surfaces

**Figure 5:**
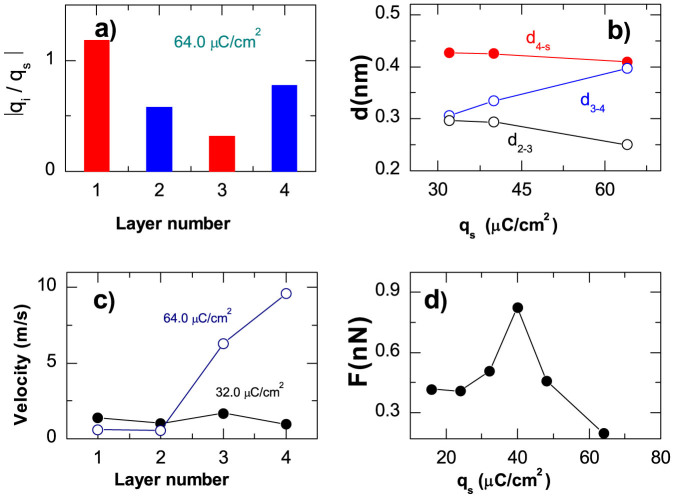
Rationalizing friction between oppositely charged surfaces. (a) Charge densities in cation (red)- and anion (blue)-rich layers normalized by the surfaces charge density, *q_s_*. (b) Charge dependence of the mean distances between the wall and the forth layer, *d*_4-*S*_ (red curve), between the fourth and third layers *d*_3–4_ (blue curve) and between the third and second layers, *d*_3-2_ (black curve). (c) Velocity profiles inside the IL film for the indicated values of *q_s_*. (d) Charge dependencies of the friction force shown at load *F_L_* = 188 MPa.

In both AFM and SFA configurations the surface charge can be controlled applying the voltage between the walls^10^,^11^,^12^,^20^, and in this case the confining walls may have opposite charges. Below we present results of simulations obtained for oppositely charged walls with the same magnitude of |*q_s_*|. The bottom mobile wall was chosen to be positively charged. In this configuration, for moderate surface charge densities (|*q_s_*| < 40 μC/cm^2^ for *F_L_* = 188 MPa), there is an even number of alternating anion and cation layers between the walls, needed to ensure the electroneutrality of the system (see Fig. 5a). For higher *q_s_* (*q_s_* > 40 μC/cm^2^) the simulation shows a crowding structure of anions at the positively charged wall. In contrast to the case of identically charged walls, here the charge distribution can be strongly asymmetric with respect to the middle of the film. We would like to recall that in our model the anion radius is twice larger than the cation radius, and this leads to a significant difference in both normal and lateral ordering of the anion layer at the positively charged wall compared to that of the cation layer at the negatively charged wall. The distance between the wall and the adjacent anion layer is larger than the distance between the other wall and the cation layer (see Figs. 3c and 5b). Hence, the corrugation of the potential energy surface felt by anions sliding above the positively charged wall is significantly lower than for cations sliding over the negatively charged wall. The difference in anion and cation sizes also influences the in-plane ordering of ions next to the wall. Because of the larger size of anions, for all values of the surface charge density the structure factor for the anion layer closest to the wall exhibits the hexagonal in-plane ordering that is determined by the electrostatic repulsion between the anions and is incommensurate with the structure of the wall.

The velocity profiles calculated for low |*q_s_*| (see Fig. 5c) demonstrate that under shear the fourth anion layer is decoupled from the wall, and the system exhibits low friction. Similarly to the case of identically charged walls, the normal ordering in confined IL strongly depends on the surface charge density (see Fig. 5b). The distance between the positively charged wall and the adjacent anion layer, *d*_4-S_, decreases with increasing |*q_s_*|, while the distance between the third and forth ionic layers, *d*_3–4_, increases with |*q_s_* |. Thus, increase of |*q_s_*| leads to a stronger coupling between the anion layer and the wall, and the overall friction force increases reaching the maximum at *q_s_* ≈ 40 μC/cm^2^ (see Fig. 5d). With further increase of the surface charge density, the slippage plane moves from the anion-wall interface towards the interior of the IL film. Hence, in accordance with experiments^12^ at high surface charges we find a super-low friction; the data shown in Fig. 5d give a friction coefficient below 0.01. The structure factor, *S*_3_(*k*), calculated for *q_s_* > 40 μC/cm^2^ indicates that the cation layer embedded between the two anion layers is in a liquid state, and this provides the superlubricity. Had the size of cation be larger than anions, the result would be same, when simultaneously changing the voltage sign. This effect has been observed in AFM experiments with ionic liquids, [BMIM]FAP and [BMIM]I, having the same cation BMIM^+^ and different anions FAP^−^ and I^−^, first of which is greater than the cation while the second one is smaller than BMIM^+^^10^. The experiments demonstrated that for [BMIM]I the response of the friction force to changes in sign of the potential is opposite to those for [BMIM]FAP. However, a direct comparison between the experimental results and simulations is problematic since in the experiments there was only one single ion layer between the two surfaces while our simulations have been performed for the multilayer cases.

All-in-all, for the IL confined between *oppositely charged walls* the simulations predict similar trends in charge dependence of friction as in the case of identically charged walls (see Figs. 3b and 5d), however in the former configuration the lower friction forces can be achieved.

In the current study we considered anions larger than cations. The results obtained for the case of cations larger than anions as in Ref. 26, which we did not show above, demonstrate a similar nonmonotonic variation of friction force with the surface charge density.

## Conclusion

We believe that the revealed mechanism of variation of the friction force with the surface charge density is generic for ILs. It allows to rationalize recent AFM friction experiments in ILs^11^,^12^. We explain an observed (i) decrease of friction with increasing applied voltage, both for negative and positive surface potentials; (ii) variation of the friction with the potential of the surface, as resulting from the voltage-induced change of composition of the layer adjacent to the surface, from cation-rich to anion-rich; (iii) the superlubricity for anion rich boundary layers. However, one must not forget that the ions considered in Refs. 11, 12 are different in shape – the cations are elongated and have asymmetric intra-ion charge distributions.

Although the analysis of the data related to specific ionic liquids may require more detailed models of ions, the key effects found should be taken into consideration:

- The friction force varies with the surface charge density through (i) its impact on the normal and in-plane ordering of the ionic liquid film and (ii) switching between anion and cation layers in contact with substrate surfaces.

- At large surface charge densities the slippage plane moves off the solid/liquid interface to the interior of the film, and thereby allows to satisfy two main requirements on an ‘ideal' lubricant -- (i) low friction coefficient and (ii) strong interaction between the ionic layers and surfaces, which prevents the “squeeze-out” of the liquid and wear under pressure. These requirements are usually considered competing, but we show that they can be satisfied simultaneously for electrified interfaces with ionic liquids.

## Methods

The molecular dynamics code, Gromacs v. 4.6.3^36^,^37^, was employed in all our computations. The long range electrostatic interactions were treated via the particle-mesh Ewald summation method (PME). All Coulomb interactions in the system have been screened by an effective dielectric constant ε* = 2.0, which accounts for electronic polarizability of the liquid. In this work, image forces have not been taken into account, which will modify electrostatic interactions between ions and ion surface interactions^27^,^38^; these effects are to be investigated elsewhere, although we do not expect changes in our conclusions. The system was coupled to a pressure barostat, P = 1 bar, via the anisotropic Berendsen algorithm, which was applied to the *x* and *z* directions, while the box length in the direction of the pulling, y, was kept constant. Periodic boundary conditions were imposed in all directions. The walls were kept at constant temperature of *T* = 600 K by rescaling the velocities of the walls atoms. This served to maintain the temperature of the IL ions, which were not coupled to an explicit thermostat.

The following values of interaction parameters have been used in the simulations: ion-ion interaction, *ε_i−i_* = 0.25 kJ/mol, ion-wall interaction *ε_i−s_* = 1.5 kJ/mol, interaction between atoms in the wall *ε_s−s_* = 500 kJ/mol, interaction between atoms of different walls *ε_s−_*_s_ = 1.0 kJ/mol and the atom sizes are set by the mixing rule 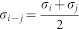.

The normal load was applied by subjecting the mobile wall to a constant force, *F_L_*, in the direction of the fixed wall. Before sliding one the walls, the whole system was equilibrated at the desired normal load for a minimum of 8 ns in order to achieve an equilibrium configuration with a well defined number of IL confined layers. Then the sliding under constant loading, *F_L_*, and shearing at constant velocity, *V_dr_*, was carried out for a further 16 ns. The integration of the equations was performed using the Leap-Frog algorithm with a time step of 2 fs. The data were collected at 6 ps time interval for further analysis.

## Author Contributions

O.Y.F., F.B., A.A.K. and M.U. designed the model, analysed the results and wrote the paper. O.Y.F. performed the simulations.
